# Pennsylvania Alumni Association of the American Veterinary College

**Published:** 1895-09

**Authors:** W. L. Rhoads

**Affiliations:** Secretary


					﻿PENNSYLVANIA ALUMNI ASSOCIATION OF THE
AMERICAN VETERINARY COLLEGE.
The first annual meeting was held at the Runnymede Club, Lansdowne,
June ii, 1895.
In the absence of Dr. Allen (who was elected Chairman at the prelimi-
nary meeting held at the office of Dr. Hoskins, February 12th), Dr. Goent-
ner was elected Chairman pro tem.
After the reading and adoption of the minutes ot the February meeting,
the merits of a State Alumni Association were discussed, thus bringing
forth its advantages to the school and the profession, as well as to the men
individually.
Officers were then nominated for the ensuing year. There being no con-,
test, the Secretary was instructed to cast the following ballot; President,
W. L. Zuill, Philadelphia. First Vice-President, Charles T. Goentner,
Bryn Mawr. Second Vice-President, U. S. G. Bieber, Kutztown. Secre-
tary-Treasurer, W. L. Rhoads, Lansdowne. Executive Committee, W.
H. Hoskins and F. S. Allen, Philadelphia; A. O’Cawley, Milton; H. J.
McClellan, Lansdowne; J. L. Bradley, Mercersburg. The Executive Com-
mittee were instructed to draft by-laws, etc.
The meeting was well attended, and after a sumptuous repast adjourned
to meet at the call of the chair.
Dr. W. L. Rhoads,
Secretary.
				

